# Elevated autistic traits and social anxiety, and reduced empathy in adult women with triple X syndrome

**DOI:** 10.1186/s11689-025-09631-7

**Published:** 2025-07-23

**Authors:** Marie-Anne Croyé, Petra Freilinger, Hendrik Jürgenlimke, Gregor Domes, Jobst Meyer

**Affiliations:** 1 Department of Neurobehavioral Genetics, University of Trier, Johanniterufer 15, 54290 Trier, Germany; 2 Genetikum, Neu-Ulm, Germany; 3 Department of Biological and Clinical Psychology, University of Trier, Trier, Germany; 4 Institute for Cognitive and Affective Neuroscience, University of Trier, Trier, Germany

**Keywords:** 47,XXX, Triple X syndrome, Trisomy X, Sex chromosome aneuploidy, Social functioning, Autistic traits, Social anxiety, Chronic stress, Stress coping mechanisms, Somatization

## Abstract

**Background:**

Triple X syndrome (TXS, 47,XXX) is a sex chromosome aneuploidy affecting females. The condition is associated with cognitive, emotional, and social challenges. While prior research has primarily focused on children, the social and psychological profile of adult women with TXS remains understudied. This study aims to provide a comprehensive assessment of these aspects in adult women with TXS compared to matched controls.

**Methods:**

A cohort of 44 women with TXS (mean age 30.5 years) was compared to 50 age- and education-matched controls (mean age 29.7 years). Standardized assessments measured verbal IQ, psychological distress, chronic stress, emotion regulation, coping mechanisms, social anxiety, empathy, autistic traits, and personality traits. Group comparisons were conducted using ANOVAs and MANOVAs, with additional χ² tests for categorical variables.

**Results:**

Depression and trait anxiety did not significantly differ between groups, though both groups exhibited notably high scores. However, a greater number of individuals in the TXS group reported elevated social anxiety and autistic traits, and reduced empathy. Moreover, there were indications of increased self-reported social tensions, personal distress, and somatization within the TXS group. No significant differences were found in personality traits, verbal IQ, chronic stress levels, and emotion regulation. Additionally, TXS participants tended to rely less on the maladaptive coping strategy of alcohol and cigarette consumption.

**Conclusion:**

Our findings underscore autistic traits, social anxiety, and reduced empathy as significant challenges for adult women with TXS. While cognitive and emotional characteristics were largely comparable to those of age- and education-matched controls, the heightened social difficulties suggest a potential benefit of targeted interventions, such as social skills training, to support affected individuals. Longitudinal studies are essential to understand the long-term progression of these challenges and to develop effective therapeutic strategies.

**Supplementary Information:**

The online version contains supplementary material available at 10.1186/s11689-025-09631-7.

## Background

Trisomy X, also known as Triple X Syndrome (TXS), is a sex chromosome aneuploidy in which females possess an additional X chromosome (karyotype: 47,XXX). While the combined incidence of gonosomal and autosomal aneuploidies is approximately 8.7 per 1000 [1], TXS is among the most common genetic syndromes in humans, with an incidence of approximately 0.65 per 1000 live-born females [2, 3]. Despite its prevalence, TXS remains underdiagnosed and underresearched, likely due to the often subtle physical symptoms and considerable variability in both physical and psychological presentation. Since the first documented case in 1959, involving an infertile woman [4], systematic research on TXS has been limited. However, as diagnoses of TXS rise with the advancement of prenatal diagnostics, research into characterizing the TXS phenotype has become increasingly urgent. Non-invasive prenatal testing (NIPT), which analyzes fetal genetic variations from maternal blood, has significantly increased the detection of chromosomal aneuploidies, including TXS [5]. Comprehensive knowledge of the clinical phenotype is essential for providing accurate genetic counseling and developing effective therapeutic and support strategies.

Physical characteristics that can occur in individuals with TXS include tall stature, epicanthal folds, clinodactyly, hypotonia in childhood, urogenital malformations, seizure disorders, motor intention disorders, as well as skeletal anomalies, dental irregularities, and delayed motor development [6].

In typically developing females, one of the two X chromosomes undergoes inactivation, though up to 15% of the genes on the inactivated X chromosome escape this process [7]. There is evidence that the additional X chromosome in TXS also undergoes X chromosome inactivation (XCI) [8], but the efficiency of XCI of the extra X chromosome remains unclear [9, 10]. Moreover, there is evidence for increased expression of X-linked genes that escape XCI in individuals with TXS compared to females with a normal karyotype [11]. Given that the X chromosome contains numerous genes essential for brain development [12], this may help explain the neurobehavioral difficulties and psychopathology observed in a proportion of individuals with TXS. The socio-cognitive and behavioral phenotype of TXS is highly variable, ranging from typical development to significant psychiatric conditions [6]. Few neuroimaging studies in children, adolescents, and adults with TXS report reductions in total brain volume and regional gray matter alterations—particularly in areas involved in executive function, language, social cognition, and motor skills—features often impaired in individuals with TXS [13, 14]. Additionally, greater amygdala and hippocampus volume has been associated with higher empathy and lower levels of anxiety and depression in women with TXS [14].

A study of military veterans who voluntarily participated in the Million Veterans Program, including 61 women with TXS (of whom less than a third had been diagnosed previously), found no significant differences in mental health conditions compared to the control group. However, due to the unique nature of the sample—military veterans—selection bias may limit the generalizability of these findings to the broader population of women with TXS [15]. In contrast, a large nationwide study from Denmark including 103 women with TXS [16], as well as a recent large-scale analysis of three international biobanks including 342 women with TXS [3], demonstrated significantly higher risks for various physical (e.g., vascular and metabolic), psychiatric, and neurodevelopmental disorders in TXS.

A previous study conducted in our department found that approximately half of the 72 girls and women with TXS displayed behavioral or social difficulties [17]. Another study by Otter et al. [18], focusing on social functioning, found similar results, with 12 out of 32 women with TXS demonstrating impaired social functioning. Moreover, this study highlighted that women with TXS, especially those with social impairments, are at increased risk for developing psychiatric disorders, including psychotic disorders, major depression, anxiety disorders, suicidality, and low self-esteem [19].

Studies on cognitive abilities have shown a reduced intelligence quotient, particularly in the verbal area [20], as well as impairments in attention control, inhibition, mental flexibility, and visual working memory [21]. Furthermore, there is evidence indicating a higher prevalence of autism symptoms or diagnoses (ASD), attention deficit hyperactivity disorder (ADHD), and psychiatric disorders such as anxiety, depression, bipolar disorder, or psychotic symptoms [2, 22].

Research into social and cognitive peculiarities has found that girls with TXS often experience difficulties with the “theory of mind” and are therefore less able to empathize with others [23]. Currently, there is no systematic assessment of empathic abilities in adults with TXS. Emotion regulation problems are also more common in this population [24], and adults with TXS appear to have reduced social skills and greater difficulties in recognizing emotions [18].

Our own prior study demonstrated that notable features in young children with TXS include attention and learning difficulties, social problems, and low self-esteem. In females aged 8 to 17 years, attention and social difficulties were more pronounced, with half of the girls experiencing challenges in school performance, behavioural issues, internalizing symptoms, and low self-esteem. The adult group, which included 17 women with TXS, exhibited increased risks for emotional instability, anxiety, depression, low self-confidence, and reduced life satisfaction, as well as difficulties in social functioning and achievement orientation [17].

While much of the research on TXS has focused on children, adolescents, and young adults—often with small, biased samples or without a control group—there is limited information on the phenotype of women with TXS in adulthood. Therefore, further research is needed to systematically characterize the TXS phenotype, particularly in adulthood.

This study aims to investigate the social phenotype of females with TXS in a larger adult sample, with a particular focus on psychometric and psychopathological characteristics using standardized psychological questionnaires. By comparing individuals with TXS to an age- and education-matched control group, we seek to systematically identify social and psychological traits that may be unique to this population, thereby contributing to a better understanding of their mental health profile.

Based on the current literature, we hypothesize that women with TXS, compared to women in the control group, exhibit more frequent depressive and (social) anxiety symptoms, lower empathy, and a higher prevalence of autistic traits. Additionally, we expect heightened somatization symptoms, reflecting a broader vulnerability to stress-related physical complaints. We also hypothesize that women with TXS have less effective emotion regulation strategies and stress coping mechanisms, leading to higher stress levels and stress-related symptoms. Furthermore, we expect women with TXS to score higher on the neuroticism scale and lower on the other four personality scales—extraversion, openness to experience, agreeableness, and conscientiousness—as this pattern is typically associated with lower self-esteem. Finally, we hypothesize that women with TXS have a lower mean verbal IQ compared to the population average.

## Subjects and methods

### Preregistration

This study was preregistered on the Open Science Framework (OSF) to enhance transparency and reproducibility. Details of the study’s design, hypotheses, and planned analyses are publicly accessible on OSF at https://osf.io/5dp3h.

### Participants

Women with TXS were recruited via the German Triple X support network (“Triplo-X-Kontaktgruppe”), human genetics specialists, and social media platforms. Eligibility required confirmation of a 47,XXX karyotype. Age- and education-matched female controls were recruited through flyers, digital newsletters, and local advertisements, with educational matching based on the highest school-leaving qualification. Participants had to be 16 years or older and fluent in German at a native level. Initially, 54 women with TXS and 64 controls expressed interest in participating; after some participants withdrew or did not meet study requirements, 44 women with TXS (aged 16–66 years) and 50 controls (aged 16–65 years) were included in the final analysis. The controls were not karyotyped, but are assumed to be 46,XX due to the low prevalence of TXS. Written informed consent was obtained from all participants, as well as from the parents of underage women. Recruitment took place between 2021 and 2023, and all participants completed the self-report questionnaires online, divided into three distinct blocks, allowing them to complete them at their own pace. This study is part of a larger research project aimed at characterizing the social phenotype of women with TXS by examining psychometric, cognitive, affective, and behavioural aspects, as well as performing neuroimaging analyses (see [14], for details on the structural neuroimaging results). The latter two were conducted with a subsample. The study protocol received approval from the University of Trier’s ethics committee.

### Measures

To assess the constructs of interest, we selected representative, psychometrically validated questionnaires. This selection was theory-driven and guided by previous research to ensure comprehensive coverage of key emotional, behavioral, and cognitive domains. Normative data based on German samples are available for several of the selected instruments, including the WST, BDI-II, STAI-T, Mini-SCL, TICS, SCI, and the NEO-FFI-30. For the remaining instruments, interpretation is based on mean scores reported in the literature.

Verbal intelligence was measured using a vocabulary test—the German *Wortschatztest* (WST) developed by Schmidt and Metzler [25], which assesses verbal ability. The test comprises 40 items, each presenting one correct word alongside five distractor words. It has demonstrated strong psychometric properties, with a split-half reliability of *r*_tt_ = 0.95 (Spearman-Brown) and an internal consistency of *α* = 0.94. The raw scores were converted into verbal IQ values using the norms provided by Schmidt and Metzler [25].

The Beck Depression Inventory-II (BDI-II) [26], adapted for the German population by Hautzinger et al. [27], was used to assess the severity of depressive symptoms. The BDI-II is a widely used 21-item questionnaire designed to measure the presence and intensity of depressive symptoms. Each item is scored on a 4-point Likert scale ranging from 0 to 3, with total scores ranging from 0 to 63. The BDI-II scores were categorized based on Hautzinger et al. [27] as follows: 0–13 for minimal symptoms, 14–19 for mild depression, 20–28 for moderate depression, and 29–63 for severe depression. The test-retest reliability was reported as *r*_tt_ = 0.78, and internal consistency ranged from *α* = 0.89 to 0.93, indicating strong reliability and consistency in measuring depressive symptoms.

The trait scale of the State-Trait Anxiety Inventory (STAI-T) [28], in its German version adapted by Laux et al. [29], was used to assess trait anxiety. The STAI-T consists of 20 items designed to measure the general tendency towards anxiety. Each item is scored on a 4-point Likert scale, with total scores ranging from 20 to 80. High anxiety scores reflect elevated levels of anxiety, with scores of 40 or above suggesting likely clinical anxiety [30]. The internal consistency was reported as *α* = 0.86 to 0.92, demonstrating strong reliability and consistency in measuring trait anxiety. The test-retest reliability was reported as *r*_tt_ = 0.77–0.90, indicating good stability over time.

The Mini-Symptom Checklist (Mini-SCL), described by Franke [31], was used to assess general psychological distress. This brief 18-item questionnaire measures a range of symptoms related to psychological distress, including “anxiety”, “depression”, and “somatization”. Each item is scored on a 5-point Likert scale, with total scores ranging from 0 to 72. The internal consistency was reported as *α* = 0.77 to 0.92, indicating satisfactory to very good reliability.

The Trier Inventory for Chronic Stress (TICS), as described by Schulz et al. [32], was used to assess chronic stress levels. This questionnaire consists of 57 items designed to measure various dimensions of chronic stress across nine subscales: “work overload”, “social overload”, “pressure to succeed”, “work discontent”, “excessive demands at work”, “lack of social recognition”, “social tensions”, “social isolation”, and “chronic worrying”. Each item is scored on a 5-point Likert scale. The TICS does not provide a total score but includes a screening scale consisting of 12 out of the 57 items, which assesses general and non-specific chronic stress levels. The internal consistency of the TICS was reported as *α* = 0.84 to 0.91, indicating good to very good reliability.

Using the questionnaire Stress and Coping Inventory (SCI) [33], consisting of 54 items, stress load, stress symptoms, and coping strategies were assessed. The SCI evaluates stress through three scales: “stress caused by uncertainty,” “stress due to excessive demands,” and “stress due to loss,” which together form a “total stress score.” These scales include 21 items, rated on a 7-point Likert scale. Additionally, the “stress symptoms” scale consists of 13 items, rated on a 4-point Likert scale. The five coping scales—“positive thinking”, “active stress coping”, “social support”, “support in faith”, and “alcohol and cigarette consumption”—each comprise four items, rated on a 4-point Likert scale. The internal consistency of the SCI ranges from *α* = 0.69 to 0.88, reflecting reliability from satisfactory to very good. For the stress scales, the Guttman split-half reliability ranges from *λ* = 0.69 to 0.82, indicating acceptable to good reliability.

The Emotions Regulation Questionnaire (ERQ) [34] assesses individuals’ emotion regulation strategies. We used the German version of the ERQ [35], which consists of 10 items, divided into two subscales: “reappraisal” and “suppression”. Each item is rated on a 7-point Likert scale. The internal consistency of the ERQ is reported to range from *α* = 0.64 to 0.76, reflecting acceptable to good reliability.

The German Brief Fear of Negative Evaluation (FNE-K) scale by Reichenberger et al. [36], was used to assess individuals’ anxiety about negative evaluations by others. This questionnaire consists of 12 items, each rated on a 5-point Likert scale. The internal consistency of the FNE-K is reported as *α* = 0.94, indicating excellent reliability. Additionally, the test-retest reliability was reported as *r*_tt_ = 0.90, reflecting good measurement accuracy.

The Liebowitz Social Anxiety Scale (LSAS), adapted into German by Stangier & Heidenreich [37], was used to assess the severity of social anxiety symptoms in both social interaction and performance situations. The questionnaire consists of 24 items, with each item rated on two separate 4-point Likert scales: one for fear/anxiety and the other measuring avoidance. In addition to a total score (ranging from 0 to 144), the LSAS assesses four factors: “fear of performance situations”, “avoidance of performance situations”, “fear of interaction situations”, and “avoidance of interaction situations”. Mennin et al. [38] established a cutoff score of 30 for the total score to diagnose social phobia, and a cutoff of 60 for generalized social phobia, which is considered a more severe and comprehensive form of social phobia. The internal consistency of the total scale is exceptionally high, with a Cronbach’s Alpha of *α* = 0.91. However, the subscales exhibit a broader range of reliability, from *α* = 0.67 to 0.81, with the subscale for performance anxiety demonstrating particularly lower consistency [39].

The German version of the Gaze Anxiety Rating Scale (GARS) [40] was used to assess individuals’ anxiety related to fear of eye gaze and avoidance of eye contact. This scale consists of 17 items, each rated on a 4-point Likert scale, measuring both fear and avoidance. In addition to a total GARS score, four subscales are assessed based on two situational categories: “fear or avoidance in everyday situations” and “fear or avoidance in situations involving high levels of social threat”. The total score has demonstrated excellent internal consistency, with *α* = 0.95, and test-retest reliability between *r*_tt_ = 0.86 and 0.72, reflecting high to moderate stability over time. Additionally, the scale shows very high split-half reliabilities, ranging from *r* = 0.91 to 0.93.

The Empathy Quotient (EQ) [41] is a validated and reliable measure of various aspects of empathy. The German version used in this study was translated by de Haen [42] and has not yet been validated. The EQ comprises 40 items, rated on a 4-point Likert scale, with total scores ranging from 0 to 80. A cutoff score of 30 serves as the threshold at which the EQ most effectively differentiates between neurotypical and autistic individuals. In addition to the EQ total score, three subscales are included: “cognitive empathy”, which refers to the ability to understand the thoughts, feelings, and perspectives of others, “emotional empathy”, which describes the capacity to attune to others’ emotions, and “social skills”, which encompass the ability to interact effectively with others [43].

The Interpersonal Reactivity Index (IRI) [44] comprises 28 items and evaluates four key dimensions related to empathy: “perspective-taking” (assesses the tendency to adopt others’ viewpoints), “fantasy” (measures identification with characters in books, movies, or plays), “empathic concern” (evaluates concern for others’ feelings and needs), and “personal distress” (assesses discomfort in challenging social situations). In this analysis, we calculated total scores by summing the scales for perspective-taking, fantasy, and empathic concern [45]. We used a German translation of the IRI, which has not been formally validated. The internal consistency of the original IRI subscales is deemed acceptable, with values ranging from *α* = 0.68 to 0.79.

A German short version of the Autism Quotient (AQ-k) [46], was used to assess traits associated with the autism spectrum. The AQ-k consists of 33 items rated on a 4-point Likert scale and measures a total score, with scores of 17 or above suggesting a likely clinical diagnosis of autism spectrum disorder, as well as three factors: “social interaction and spontaneity”, “fantasy and imagination”, and “communication and reciprocity”. The AQ-k has proven to be a reliable tool, with internal consistency ranging from *α* = 0.65 to 0.87 for the subscales and *α* = 0.79 for the total score. It has also been shown to effectively differentiate between individuals on the autism spectrum and those with typical development.

The German short version of the NEO-Five Factor Inventory (NEO-FFI-30) [47] was employed to assess the Big Five personality traits: “neuroticism”, “extraversion”, “openness to experience”, “agreeableness”, and “conscientiousness”. The NEO-FFI-30 consists of 30 items, rated on a 5-point Likert scale and yields scores for each of the five personality dimensions. The scales demonstrate acceptable internal consistencies ranging from *α* = 0.67 to 0.81.

### Analysis

Demographic sample characteristics were compared using χ² tests or t-tests. For total scores, analyses of variance (ANOVAs) were conducted. To compare the subscale profiles between groups, separate multivariate analyses of variance (MANOVAs) were performed, followed by univariate ANOVAs to further explore effects on the specific subscales. Statistical tests were carried out using SPSS for Windows, Version 28. The significance level for all tests was set at *α* = 0.05. To correct for multiple comparisons in the MANOVAs/ANOVAs, a corrected significance threshold was applied. A total of 19 scales were included in the calculation of the effective number of independent tests (M_eff_), using the method proposed by Li and Ji [48]. This number arises because two of the 14 questionnaires used (NEO-FFI and ERQ) do not provide a single total score and were therefore represented by their respective subscales. Additionally, the “personal distress” subscale of the IRI was treated separately, as it is not considered a direct measure of empathy [43]. Based on a principal component analysis of the correlation matrix, the M_eff_ was approximately 10.49. Accordingly, the significance threshold was adjusted using a Bonferroni-type correction (*α* = 0.05/10.49 ≈ 0.005). In addition, five χ² tests were conducted to examine group differences based on published cutoff scores. For these tests, a separate Bonferroni correction was applied, resulting in a significance threshold of *α* = 0.05/5 = 0.01.While primary interpretations are based on the corrected thresholds, uncorrected results (*α* = 0.05) are additionally reported to allow for comprehensive evaluation and ensure transparency.

## Results

Table 1 presents demographic characteristics of the triple X and control groups. The triple X group did not show significant differences from the control group in age, education, verbal IQ, and marital status. Triple X females demonstrated a lower BMI (*p* = 0.001; Cohen’s *d* = 0.70) and greater height (*p* = 0.000007; Cohen’s *d* = 0.98). They were more often childless (*p* = 0.045; *Φ* = 0.21) and non-smokers (*p* = 0.002; *Φ* = 0.32) and fewer women in the triple X group consumed alcohol (*p* = 0.039; *Φ* = 0.21). Nearly three times as many women with TXS had at least one physical health condition compared to the control group (*p* = 0.003; *Φ* = 0.31).

**Table 1:** Demographic characteristics of women with TXS and controls

	TXS (*n* = 44)		Controls (*n* = 50)		Statistical Test		
	***M***	***SD***	***M***	***SD***	***t***	***p***	***d***
Age	30.5	12.2	29.7	11.5	0.301	0.764	0.06
Education^1^	11.0	1.7	11.1	1.7	−0.232	0.817	−0.05
Verbal IQ (WST)	98.6	14.4	97.9	12.2	0.265	0.792	0.06
BMI	22.0	4.6	25.6	5.5	3.377	0.001	0.70
Height (cm)	1.74	0.09	1.67	0.06	4.650	0.000007	0.98
	***%***		***%***		***χ2***	***p***	***Φ***
Married^2^	22.7		14.0		1.203	0.273	0.11
Parental status^3^	15.9		34.0		4.029	0.045	0.21
Smoking^4^	22.7		54.0		9.590	0.002	0.32
Alcohol consumption^4^	38.6		60.0		4.273	0.039	0.21
Morbidity^5^	44.2		16.0		8.914	0.003	0.31

Notes: *WST* Vocabulary Test (‘Wortschatztest’); *BMI* body mass index

^1^years in school; ^2^married vs. unmarried/widowed/divorced; ^3^at least one child vs. no; ^4^regularly or occasionally vs. no; ^5^at least one physical morbidity vs. no

20 (45.5%) of the triple X females were diagnosed prenatally, 22 (50%) postnatally, and 2 (4.5%) did not provide information regarding the timing of diagnosis. Among the postnatally diagnosed participants in our sample, 8 (36%) received their diagnosis during childhood or adolescence, while 12 (55%) were diagnosed in adulthood. In 2 cases (9%), age at diagnosis was not reported. Most diagnoses occurred in the context of investigations for medical concerns, including developmental delays (e.g., language impairment, epilepsy), unexplained physical symptoms (e.g., abdominal pain, swelling), and family history of genetic or neurodevelopmental conditions (e.g., autism, affected twin). Several diagnoses were incidental findings during fertility assessments or genetic testing for other suspected conditions (e.g., muscle disease, suspected intersex condition, Ehlers–Danlos syndrome, fetal anomaly). Postnatally diagnosed women were older (mean age = 33.1 vs. 26.4 years; *p* = 0.044; Cohen’s *d* = − 0.63) and more likely to be married (26.4% vs. 10.0%; *p* = 0.045; *Φ* = − 0.31) compared to prenatally diagnosed women. A full comparison of demographic data between prenatally and postnatally diagnosed women with TXS is presented in Table S1. No significant differences were observed between prenatally and postnatally diagnosed females with respect to any of the scales or subscales of the questionnaires. However, the direction of group differences generally tended toward higher scores in the prenatally diagnosed group, particularly in domains such as depression, stress, and social anxiety (see Table S2).

**Fig. 1:**
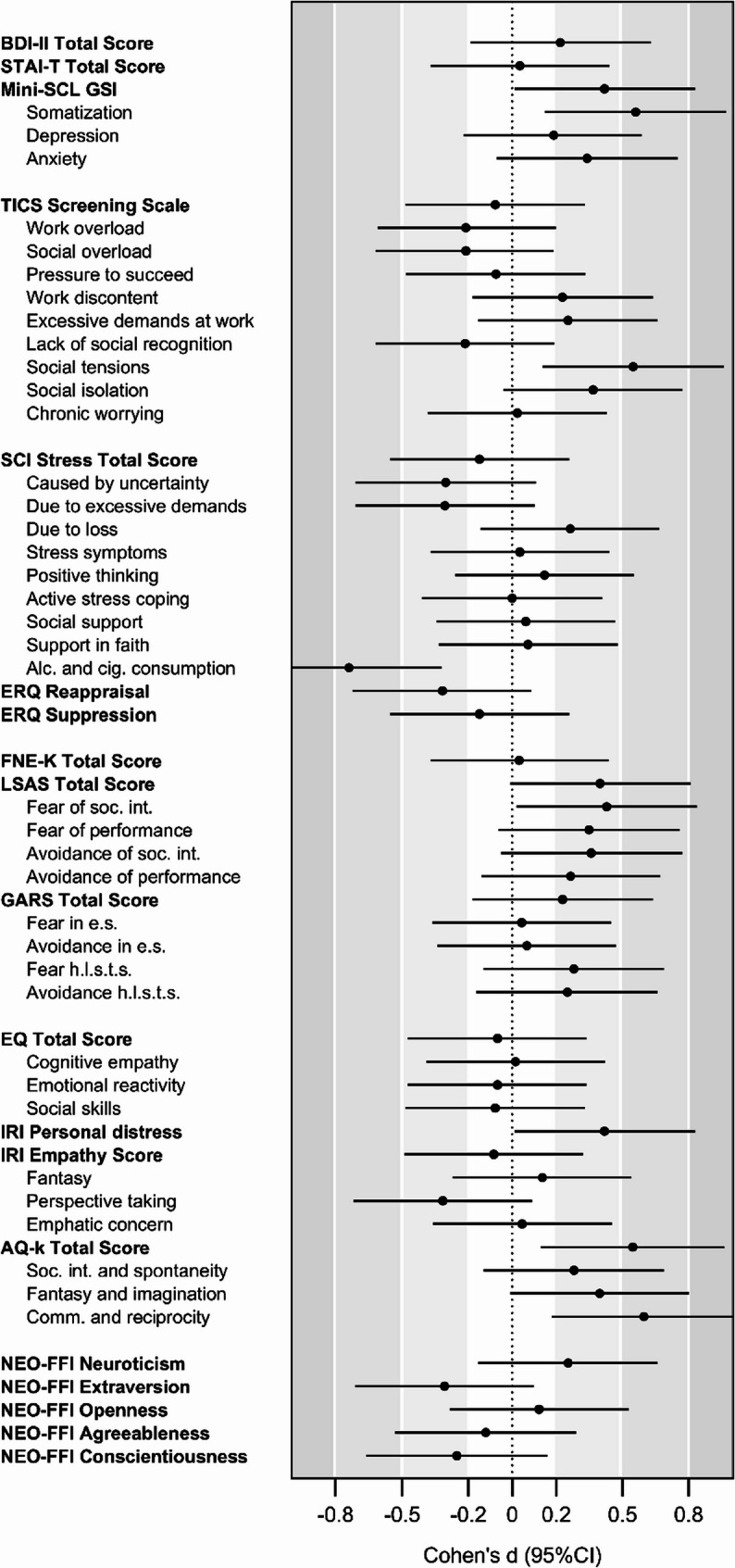
Standardized effect sizes (Cohen’s *d*) for psychometric scales comparing women with TXS and controls. Each point represents a standardized effect size, with horizontal lines indicating 95% confidence intervals (CIs). Grey areas represent small, medium, and large effect size ranges according to Cohen [49]. Positive values refer to higher scores in the TXS group compared to controls Notes: *BDI-II* Beck Depression Inventory II; *STAI-T* Trait scale of the State-Trait Anxiety Inventory; *Mini-SCL* Mini Symptom Checklist; *GSI* Global Severity Index; *TICS* Trier Inventory for Chronic Stress; *SCI* Stress and Coping Inventory; *ERQ *Emotion Regulation Questionnaire; *Alc.* Alcohol; *cig.* cigarettes; *FNE-K* Fear of Negative Evaluation Scale– short version; *LSAS* Liebowitz Social Anxiety Scale; *soc. int.* social interaction; *GARS* Gaze Anxiety Rating Scale; *e.s.* everyday situations; *h.l.s.t.s.* high level social threat situations; *EQ* Empathy Quotient; *IRI* Interpersonal Reactivity Index; *AQ-k* Autism Spectrum Quotient short version; *comm.* communication; *NEO-FFI* NEO-Five-Factor Inventory

**Fig. 2:**
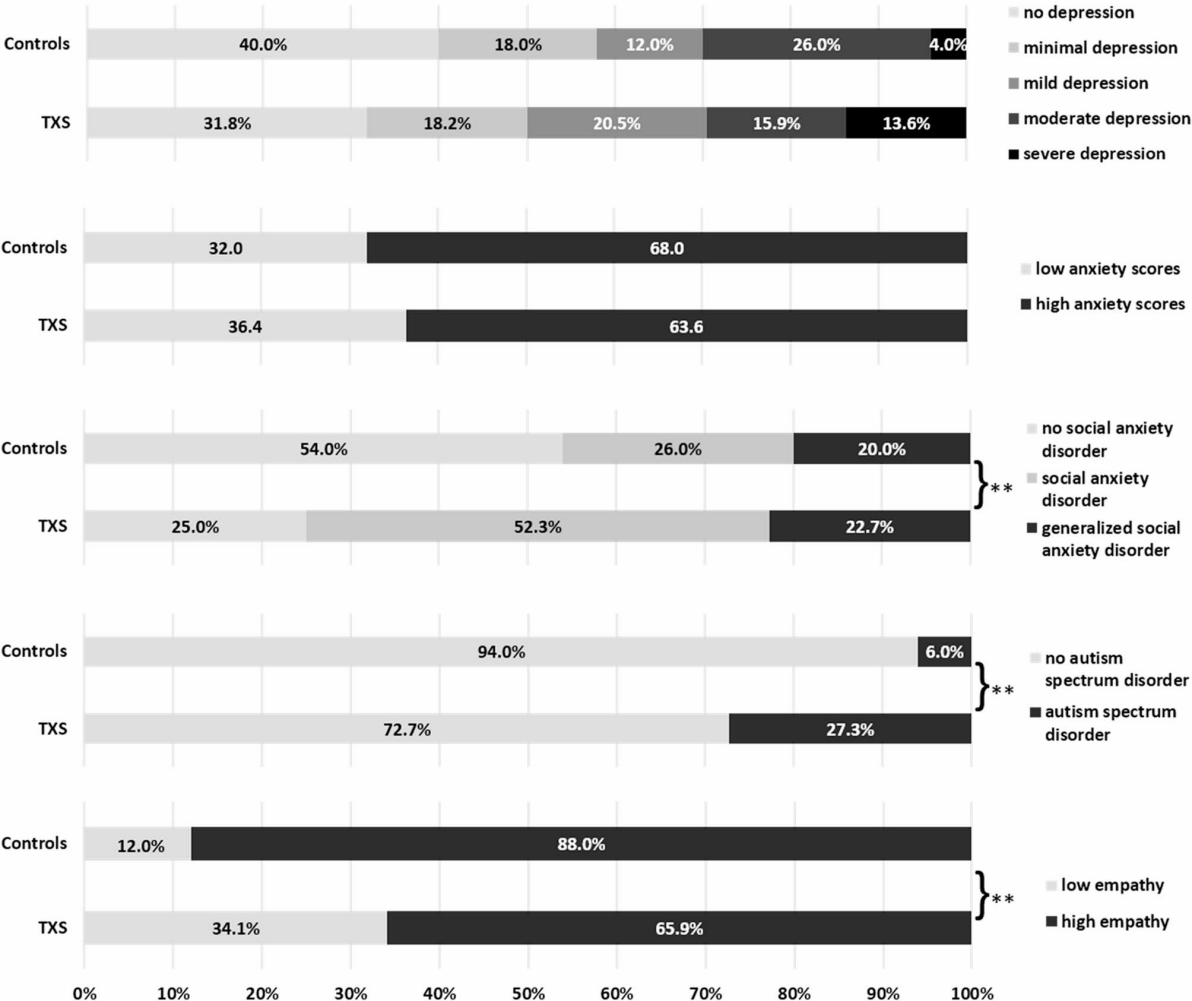
Percentage of women classified into varying severity categories based on published cutoff scores for depression (BDI-II), anxiety (STAI-T), social anxiety (LSAS), autism spectrum disorder (AQ-k), and empathy (EQ) in the TXS and control groups. Group differences were assessed using χ² tests. **: *p* ≤ 0.01

Regarding psychometric and psychopathological characteristics assessed using MANOVA/ANOVA to compare mean group differences, no significant effects emerged across the majority of scales when applying the corrected significance threshold of *α* = 0.005 to account for multiple comparisons. For completeness and transparency, results based on the conventional *α* = 0.05 level are also reported to allow for a more comprehensive interpretation. While key findings are described in the text, full results are available in Table S3, and standardized effect sizes (Cohen’s *d*) across all scales, illustrating the magnitude and direction of group differences, are presented in Fig. 1. Additionally, significant group differences in the distribution of severity levels across several characteristics were observed based on predefined cutoffs and analyzed using χ² tests with an adjusted significance threshold of *α* = 0.01. These results are illustrated in Fig. 2.

With respect to psychopathological symptoms, no significant differences were observed between the groups in terms of depression and anxiety, as assessed by the BDI-II, STAI-T, and the Mini-SCL subscales “depression” and “anxiety”. Similarly, the distribution of depression and anxiety severity levels, based on BDI-II and STAI-T cutoffs, did not significantly differ between groups (BDI-II: *χ*^*2*^ = 5.156; *p* = 0.272; *Φ* = 0.23; STAI-T: *χ*^*2*^ = 0.198; *p* = 0.656; *Φ* = 0.05) (see Fig. 2).

Regarding the Mini-SCL Global Severity Index, a comprehensive measure of overall psychological distress, the triple X group scored higher than the control group at the uncorrected significance level (*p* = 0.044; *η²p* = 0.04). Follow-up analyses of the subscales indicated that this pattern was primarily driven by elevated scores in somatization (*p* = 0.008; *η²p* = 0.08). These findings should be interpreted with caution, as the overall MANOVA across the three Mini-SCL subscales did not reach statistical significance (*p* = 0.063; *η²p* = 0.08), and the follow-up analyses were exploratory in nature.

In terms of chronic stress and coping mechanisms, neither the TICS screening scale nor the SCI total score revealed significant group differences. Nonetheless, the MANOVA for the TICS subscales indicated a statistically significant overall group effect at the uncorrected level (*p* = 0.032; *η²p* = 0.19). Follow-up analyses showed that the triple X group scored higher on the TICS subscale “social tensions” (*p* = 0.010; *η²p* = 0.07). The MANOVA for the SCI stress subscales also reached significance and survived correction for multiple comparisons (*p* = 0.004; *η²p* = 0.16). However, none of the individual SCI subscales showed significant group differences in the univariate analyses. As group means varied in direction, the observed multivariate effect may be attributable to this divergence rather than a consistent group difference across subscales. The MANOVA analysis of the SCI coping scales revealed a significant global difference at the uncorrected level (*p* = 0.035; *η²p* = 0.13). In the follow-up analyses, no significant effects for the four adaptive coping strategies were found. However, the triple X group demonstrated a significantly lower reliance on the maladaptive coping strategy “alcohol and cigarette consumption” compared to the control group (*p* = 0.0005; *η²p* = 0.12). Regarding emotion regulation strategies, as measured by the ERQ, no significant effects were found for either the adaptive strategy “reappraisal” or the maladaptive strategy “suppression”.

Concerning aspects of social anxiety, there were no significant differences between groups in terms of fear of negative evaluation or fear of eye contact, as indicated by non-significant effects in both the FNE-K total score and the GARS total score and subscales. While the LSAS total score yielded a p-value just above the conventional threshold (*p* = 0.057; *η²p* = 0.04), the difference was not statistically significant. Similarly, the MANOVA for the LSAS subscales did not indicate a significant multivariate group effect. Furthermore, significant group differences were found in the prevalence of social anxiety disorder based on specific LSAS total score cutoffs. In the triple X group, 52.3% met criteria for social anxiety disorder (controls: 26%), and an additional 22.7% had generalized social anxiety disorder (controls: 20%), showing a significant difference between groups at the corrected significance level (*χ*^*2*^ = 9.169; *p* = 0.010; *Φ* = 0.31) (see Fig. 2).

In terms of empathy, no significant group differences were observed in the EQ and IRI total empathy scores or in the MANOVA conducted on their respective subscales. However, a separate univariate analysis of the IRI “personal distress” subscale—which is not included in the IRI total score—revealed a significant difference at the uncorrected level: women in the triple X group reported higher levels of personal distress compared to the control group (*p* = 0.039; *η²p* = 0.05). Additionally, the proportion of individuals classified as having reduced empathy, based on an EQ total score cutoff, differed significantly between groups. In the triple X group, 34.1% of participants fell below this threshold, compared to 12% in the control group (*χ*^*2*^ = 6.583; *p* = 0.010; *Φ* = 0.26) (see Fig. 2). This group difference remained statistically significant after correction for multiple comparisons.

The triple X group also exhibited more autistic traits, as evidenced by a higher AQ-k total score (*p* = 0.009; *η²p* = 0.07) and a global effect in the MANOVA (*p* = 0.034; *η²p* = 0.09). These effects were statistically significant at the uncorrected level. Univariate analyses further revealed a significant difference on the AQ-k subscale “communication and reciprocity” (*p* = 0.003; *η²p* = 0.09). The prevalence of autism spectrum disorder, as defined by a specific AQ-k total score cutoff, differed significantly between groups. In the triple X group, 27.3% of participants met the cutoff criteria, whereas 6% of participants in the control group did so (*χ*^*2*^ = 7.897; *p* = 0.005; *Φ* = 0.29) (see Fig. 2). This effect remained statistically significant after correction for multiple comparisons.

No significant differences between groups were observed for the five major personality traits—neuroticism, extraversion, openness to experience, agreeableness, and conscientiousness—as assessed by the NEO-FFI.

## Discussion

In the present study, we systematically examined the social and psychological phenotype of adult women with TXS. We compared a relatively large cohort of 44 women with TXS to a well-matched control group based on sex, age, and level of education. We assessed key areas including demographic factors, verbal IQ, psychopathology, emotion regulation, coping mechanisms, social anxiety, empathy, and autistic traits. The findings offer a nuanced understanding of their mental health profile, aligning with prior research while also providing novel insights. Beyond group-level comparisons, we examined whether the timing of diagnosis (prenatal vs. postnatal) within the TXS group was related to differences in psychological outcomes. Although no statistically significant differences were found between prenatally and postnatally diagnosed females in our sample, we observed a general tendency toward higher scores of internalizing symptoms (e.g., depression, stress, social anxiety) in the prenatally diagnosed group. This somewhat contrasts with findings by Wigby et al. [50], who reported better overall psychosocial outcomes among prenatally diagnosed females compared to those diagnosed postnatally. Possible explanations for this discrepancy include differences in sample characteristics, assessment methods, or the timing and context of diagnosis. Our results suggest that early diagnosis does not necessarily provide a straightforward protective effect against psychological symptoms. Further studies with larger cohorts are needed to clarify the impact of prenatal diagnosis on mental health outcomes in individuals with TXS.

The lack of significant differences in age and education supports the validity of our matching process. Unlike previous studies, which rarely accounted for education, we matched participants on this factor due to its profound influence on cognitive, social, and psychological outcomes, which may contribute to the differing findings. However, it should be noted that educational attainment is not independent of cognitive and psychological factors and can also be influenced by them. Thus, matching on education may reduce observed group differences by controlling for a variable that partly reflects the outcome itself.

The verbal IQ of both groups was slightly below 100, with no significant difference between the triple X and control groups. This finding contrasts with previous studies that reported reduced IQ scores around 80 to 85 in children, adolescents, and relatively young women with TXS [51–55], indicating mild cognitive impairments. Our results did not show the expected lower verbal IQ in the TXS group, which may partly reflect differences in study design, such as the use of the WST—a simplified method for estimating selectively verbal IQ. However, in a parallel study with a subsample, we used the more comprehensive CFT-20 to measure general intelligence, which yielded similar results [14]. A critical factor that likely contributes to this discrepancy is ascertainment bias. Our sample consisted of adults with TXS who were capable of completing an extensive online self-report battery, suggesting that participants with relatively higher cognitive functioning may have been more inclined or able to participate. This potential selection bias must be taken into account when interpreting the absence of significant group differences in IQ. While differences in sample characteristics—such as age—may also play a role, and while it is conceivable that cognitive deficits seen in childhood might not persist into adulthood, these explanations are more speculative. Furthermore, earlier studies may have captured effects of domain-specific challenges, such as attention difficulties, which are more prevalent in individuals with TXS due to the higher rate of ADHD [22]—a factor we did not assess in our study.

In addition to cognitive and psychological assessments, we also observed distinct patterns in physical health and lifestyle factors. Our results align with previous small studies [51, 56] and the Million Veteran Program study of 61 American women with TXS [15], which reported a taller stature. Unlike the Veteran study, which found no BMI differences and classified both groups as overweight, our study found both groups to have relatively normal weight, with TXS women having a significantly lower BMI. This discrepancy may reflect cultural differences in diet and lifestyle factors. An American study from 1990 noted a tendency toward being underweight in TXS women, which may partly reflect lower BMI levels in the general U.S. population at that time.

The TXS group was more often childless, a finding that aligns with earlier studies noting reduced relative reproductive rate, lower fertility, and increased incidence of premature ovarian failure in women with TXS [2, 16, 57–59]. Nearly three times as many women with TXS had at least one physical health condition compared to controls. Prior large-scale studies have identified an increased occurrence of a wide variety of diseases in the TXS population [3, 16]. Our findings reaffirm the heightened health burden in this group and highlight the need for routine medical screening and preventive care in women with TXS.

Additionally, there was a lower prevalence of smoking and alcohol consumption in the TXS group. To the best of our knowledge, apart from the Million Veteran Program Study, which found no significant differences, this aspect has not yet been investigated in TXS. Both alcohol and nicotine use or dependence, respectively, are highly comorbid [60], genetically influenced complex conditions [61] and are generally more prevalent among men than among women [62]. Genes that escape X-inactivation on the X chromosome and are therefore more highly expressed in women with TXS may contribute to phenotypic variability in such traits, though their specific roles remain to be clarified.

In terms of psychometric assessment, although only cutoff-based categorical comparisons survived correction for multiple comparisons, uncorrected findings are also discussed in relation to their effect sizes, due to their potential relevance. Among these, the TXS group showed higher levels of self-reported autistic traits, particularly in communication and reciprocity, as well as a greater percentage of individuals exceeding the cutoff for a likely clinical ASD diagnosis, both with a medium effect size. Although meeting AQ-k criteria is not equivalent to a clinical diagnosis of ASD, the AQ-k is considered an effective tool for differentiating individuals on the autism spectrum from those with typical development [46]. Our findings corroborate earlier studies indicating elevated rates of ASD in children and young adults with the karyotypes XXX, XXY, XYY, and X [2, 23, 50]. Van Rijn et al. [23] demonstrated that children with an extra X chromosome exhibited fewer autism symptoms than those with ASD but showed increased social anxiety compared to controls, a pattern not observed in ASD children, highlighting differences in social reflective abilities. Similarly, the findings in our adult sample revealed a greater prevalence of likely social anxiety disorder (SAD) in the TXS group, as determined by specific LSAS total score cutoffs. These findings align with prior research indicating impaired social functioning in adult women with TXS [18]. Furthermore, Otter et al. [55] identified a prevalence of generalized anxiety disorder (GAD) of 20.6% in their sample, compared to 3.2% in non-education-matched controls. This aligns with our own prevalence rate of 22.7%, although our controls also showed a relatively high prevalence of 20.0%. As previously discussed, this elevated rate in our control group may be due to education-matching, since social anxiety has also been correlated with lower socio-economic status [63]. Interestingly, there were no significant differences in terms of self-reported fear of negative evaluation and gaze anxiety, both of which are positively correlated with social anxiety [40]. This suggests that while some aspects of social anxiety appear to be elevated in the TXS group, these specific dimensions may not be as strongly affected.

In partial contrast to our initial hypothesis of reduced empathy in TXS—which was based on a previous study on children with TXS [23], we found no significant differences in overall empathy scores or on the IRI subscales “fantasy”, “perspective taking”, or “empathic concern”. However, the TXS group exhibited elevated scores on the subscale “personal distress”, with a moderate effect size. As discussed by Lawrence et al. [43] this subscale is not considered a direct measure of empathy, as it is self-oriented rather than other-oriented, assessing personal anxiety and discomfort when witnessing another’s distress. Personal distress is linked to social withdrawal [64] and social anxiety [65]—traits that, as described above, were observed more frequently in individuals with TXS in our study and in previous research. Additionally, the proportion of individuals classified as having low empathy based on their self-report differed significantly between groups, with nearly three times as many individuals in the TXS group falling below the EQ cutoff compared to controls, with a moderate effect size. This suggests that while group differences may not be apparent in the total empathy scores, a subset of individuals with TXS reports particularly low empathy levels. In contrast to ASD, which is typically associated with deficits in cognitive empathy and, to a lesser extent, affective empathy [55], TXS shows a more varied pattern of empathic traits.

These social difficulties, particularly the elevated rates of social anxiety, autism spectrum traits, personal distress, and reduced empathy are likely contributors to the increased risk of psychological disorders observed in women with TXS in prior studies. In the following section, we will explore the broader emotional challenges faced by this group, focusing specifically on depression, anxiety, somatization, and chronic stress. No significant differences were found for general measures of depression (BDI-II), trait anxiety (STAI-T), or neuroticism (NEO-FFI), contrasting with previous findings reporting elevated symptoms in individuals with TXS [2, 13, 16, 17, 19, 50]. However, scores in both groups were notably high compared to normative mean values reported in manuals or validation studies [27, 29, 47]. Davis et al. [15], in their Veteran Study, found similar results, with no significant differences between groups and a relatively high prevalence of anxiety and depression in both cases and controls, which they attributed to possible effects of military service. Notably, their study also reported similar education levels in both groups, aligning with our study, in which participants were matched for education level.

Depression and anxiety, often grouped as emotional disorders, share etiologies and vulnerability factors such as trait anxiety and neuroticism, which were considered as synonymous by some authors, as both describe a predisposition toward experiencing negative emotions like worry and distress [66, 67]. These elevated scores across both groups may reflect broader societal trends, such as rising emotional distress and disorder prevalence, exacerbated by the COVID-19 crisis [68–70]. These trends are particularly pronounced in women, younger individuals, and those of lower socio-economic status—characteristics reflected in our sample. Given that women with TXS often have lower socio-economic status [71], the inclusion of education-matched controls—a rarely implemented approach in prior TXS studies—may partly explain the high scores observed even in the control group.

While no significant differences in psychological distress, as measured by the BDI-II, STAI-T, and the neuroticism scale of the NEO-FFI, were observed, the TXS group showed higher Mini-SCL GSI scores, with a moderate effect size, indicating greater psychological distress, primarily driven by elevated somatization scores. Differences in the sensitivity and focus of different assessment tools may explain these discrepancies, as somatic symptoms are not assessed by the three questionnaires mentioned above. The Mini-SCL results align with earlier research, including our previous study, where increased scores for somatic complaints were also observed [17, 19]. Somatization occurs when individuals express psychological distress through physical symptoms rather than directly experiencing it emotionally. Together, these findings further corroborate the elevated psychological distress in individuals with TXS, highlighting the need for therapeutic interventions aimed at enhancing emotional processing and expression, thereby reducing the reliance on somatic manifestations of distress.

To our knowledge, this is the first study to systematically investigate chronic stress in women with TXS. We examined various facets and sources of chronic stress, as well as coping and emotion regulation strategies, using three diagnostic tools: the TICS, SCI, and ERQ. Notable differences were observed in social tensions, with a medium effect size, as women with TXS exhibited higher scores, highlighting challenges in interpersonal contexts that may contribute to their stress experience. These findings are consistent with prior research indicating that social functioning is a key area of difficulty for individuals with TXS [17–19] and further underscore the elevated levels of social anxiety and personal distress identified in this study, emphasizing the significant challenges individuals with TXS face in navigating interpersonal contexts. No significant differences were observed across the remaining TICS and SCI stress subscales, including all work- and performance-related stress scales, suggesting that elevated social stress might be more domain-specific rather than a generalized stress response.

While adaptive coping strategies did not differ significantly between groups, the TXS group demonstrated significantly lower reliance on the maladaptive strategy of alcohol and cigarette consumption, with a medium to large effect size. This finding aligns with the previously discussed lower prevalence of smoking and alcohol use in this group.

Regarding emotion regulation strategies, no differences emerged for either reappraisal or suppression, suggesting that the elevated social stress observed in the TXS group is not necessarily attributable to specific deficits in emotion regulation or adaptive coping strategies. However, it is important to note that self-report measures of coping strategies, like those used in this study, can be limited by response biases and may oversimplify the complexity of coping mechanisms, potentially affecting the accuracy of observed patterns [72].

Our previous findings highlighted low self-esteem and low self-confidence in individuals with TXS [17]. Given the relationship between self-esteem and personality—where low self-esteem has been associated with higher neuroticism and lower scores in extraversion, openness to experience, agreeableness, and conscientiousness [73]—and the heritability of both traits (with approximately 30% of the variance attributed to genetic factors; [74–76]), we hypothesized that women with TXS would exhibit this personality profile. Contrary to this expectation, no significant differences in personality traits were found between groups. Notably, self-esteem was not assessed in this study, so it remains unclear whether the results of our previous study regarding self-esteem would have been corroborated in our sample.

A central limitation of this study lies in the use of self-report measures, which may be subject to biases such as social desirability or inaccurate self-perception. This is particularly relevant when assessing sensitive topics such as social anxiety, coping strategies, and emotion regulation. Additionally, while the study carefully matched groups based on age and education, other potentially important socio-economic factors, such as employment status, income, and housing conditions, were not systematically assessed. These factors could significantly influence the psychological well-being and social challenges of the participants and should be more thoroughly considered in future studies. Moreover, comorbid conditions, such as ADHD, were not assessed in this study, despite the higher prevalence of ADHD in individuals with TXS [2, 22], which could potentially affect cognitive and emotional outcomes. The cross-sectional design of the study also limits the ability to draw causal conclusions about the relationship between TXS and the observed psychological and social phenomena. Longitudinal studies would provide valuable insights into how these traits evolve over time. Additionally, the recruitment of participants primarily from a self-help group may introduce a bias toward individuals who experience more pronounced symptoms, as those with fewer challenges might be less likely to seek support from such groups. This could lead to an overrepresentation of more severe cases in the sample, skewing the results. At the same time, participation in a study requires a certain level of willingness, courage, and commitment. Therefore, it is conceivable that women with TXS who are more severely affected may not have been willing or able to fill out extensive questionnaires and, as a result, were not included in the study. In this context, findings from the large Danish iPSYCH2015 case-cohort study [2] are particularly relevant: the study found that all examined sex chromosome aneuploidy (SCA) karyotypes (X, XXX, XXY, XYY) were linked to an increased risk of psychiatric disorders, which was similarly present in individuals both with and without a clinical SCA diagnosis. This suggests that psychiatric vulnerabilities in TXS and other SCAs occur independently of clinical detection and similarly affect undiagnosed populations. Lastly, the education matching process itself could introduce a bias, as participants in the control group who were matched for education might come from specific socio-economic backgrounds, which could influence their responses and outcomes. Notably, women with TXS are associated with a lower socio-economic status [71], which typically correlates with lower educational attainment. As a result, the control group participants, who were matched based on education, may have below-average educational levels and therefore may not fully represent the general population. This could explain why the control group often exhibited higher scores compared to normative data, potentially distorting the comparison. Moreover, since education can act as a proxy for cognitive ability, matching on this variable may inadvertently mask true group differences, highlighting a methodological trade-off between controlling confounds and potentially underestimating effects.

## Conclusion

While previous TXS studies mainly focused on children, especially regarding autism and empathy, this study extends the understanding to an adult, relatively unbiased sample, providing nuanced insights into the social and psychological phenotype of adult women with TXS and shedding light on the complexities of their emotional and social experiences. While cognitive and psychological traits such as verbal IQ, personality, trait anxiety, depression, generalized chronic stress, emotion regulation and most coping mechanisms did not significantly differ from education-matched controls, the elevated proportion of individuals in the TXS group with self-reported social anxiety, reduced empathy, and autistic traits, as well as indications of elevated levels of self-reported social tensions, personal distress, and somatization, underscore the need for targeted interventions including social skills training and psychotherapeutic approaches aimed at improving interpersonal functioning—to address these challenges. Future studies should investigate these identified aspects longitudinally to improve clinical care and support strategies for women with TXS. In this context, longitudinal research on adult women with TXS who were assessed in childhood or adolescence would be valuable. The aim would be to assess whether social problems and anxiety symptoms persist into adulthood or if individuals without early symptoms remain unaffected later in life.

## Supplementary Information

Supplementary Material 1.

## Data Availability

The dataset used and analysed for the current study is available from the corresponding author on reasonable request.
