# Supplementary material for: Elevated autistic traits and social anxiety, and reduced empathy in adult women with triple X syndrome
Source: J Neurodev Disord. 2025 Jul 23;17:41. doi: 10.1186/s11689-025-09631-7 (PMC12285195; doi:10.1186/s11689-025-09631-7)
Supplement: Supplementary file 1 — Supplementary Material 1. [file 11689_2025_9631_MOESM1_ESM.docx]

**Supplementary Materials**

**Elevated autistic traits and social anxiety, and reduced empathy in adult women with triple X syndrome**

Marie-Anne Croyé^1^, Petra Freilinger^2^, Hendrik Jürgenlimke^1^, Gregor Domes^3,4^, Jobst Meyer^1,4^

^1^ Department of Neurobehavioral Genetics, University of Trier, Trier, Germany

^2^ Genetikum, Neu-Ulm, Germany
^3^ Department of Biological and Clinical Psychology, University of Trier, Trier, Germany

^4^ Institute for Cognitive and Affective Neuroscience, University of Trier, Trier, Germany

**Table S1**. Demographic characteristics of women with TXS diagnosed prenatally and postnatally

|  | prenatal (n=20) | | | postnatal (n=22) | | |  | | Statistical Test | | | |  |
| --- | --- | --- | --- | --- | --- | --- | --- | --- | --- | --- | --- | --- | --- |
|  | ***M*** | ***SD*** |  | | ***M*** | ***SD*** | |  | | ***t*** | ***p*** | ***d*** | |
| Age | 26.4 | 7.8 |  | | 33.1 | 12.6 | |  | | 2.039 | 0.044 | -0.63 | |
| Education^1^ | 10.6 | 1.7 |  | | 11.5 | 1.8 | |  | | -1.609 | 0.116 | -0.50 | |
| Verbal IQ (WST) | 96.0 | 16.1 |  | | 101.1 | 12.7 | |  | | 1.128 | 0.270 | 0.35 | |
| BMI | 21.4 | 4.4 |  | | 22.8 | 4.9 | |  | | -0.968 | 0.339 | -0.30 | |
| Height (cm) | 1.75 | .08 |  | | 1.74 | .09 | |  | | 0.108 | 0.914 | 0.03 | |
|  | **%** | |  | | **%** | | |  | | ***χ2*** | ***p*** | ***Φ*** | |
| Married^2^ | 10.0 | |  | | 36.4 | | |  | | 4.014 | 0.045 | -0.31 | |
| Parental status^3^ | 5.0 | |  | | 22.7 | | |  | | 2.689 | 0.101 | 0.25 | |
| Smoking^4^ | 25.0 | |  | | 13.6 | | |  | | 0.877 | 0.349 | -0.15 | |
| Alcohol consumption^4^ | 40.0 | |  | | 31.8 | | |  | | 0.305 | 0.580 | -0.09 | |
| Morbidity^5^ | 30.0 | |  | | 54.5 | | |  | | 3.304 | 0.192 | 0.28 | |

Notes: *WST* Vocabulary Test (‘Wortschatztest’); *BMI* body mass index

^1^ years in school; ^2^ married vs. unmarried/widowed/divorced; ^3^ at least one child vs. no; ^4^ regularly or occasionally vs. no; ^5^ at least one physical morbidity vs. no

**Table S2.** Group differences in psychometric and psychopathological characteristics of women with TXS diagnosed prenatally and postnatally

|  | prenatal (n=20) | | postnatal (n=22) | | |  | **MANOVA**/ANOVA | | |  |  |
| --- | --- | --- | --- | --- | --- | --- | --- | --- | --- | --- | --- |
|  | ***M*** | ***SD*** |  | ***M*** | ***SD*** |  | ***F*** | ***p*** | ***η^2^p*** |  | ***d* [95 %-CI]** |
| **BDI-II Total Score** | 16.3 | 12.1 |  | 12.7 | 9.9 |  | 1.074 | 0.306 | 0.03 |  | 0.32 [-0.29, 0.93] |
| **STAI-T Total Score** | 46.2 | 11.8 |  | 44.6 | 11.9 |  | 0.191 | 0.664 | 0.01 |  | 0.13 [-0.47, 0.74] |
| **Mini-SCL GSI** | 18.1 | 14.3 |  | 18.0 | 14.1 |  | 0.001 | 0.982 | 0.00 |  | 0.01 [-0.60, 0.61] |
| **Mini-SCL Subscales** |  |  |  |  |  |  | **1.428** | **0.250** | **0.10** |  |  |
| Somatization | 5.7 | 5.5 |  | 7.2 | 5.1 |  | 0.870 | 0.356 | 0.02 |  | -0.28 [-0.89, 0.33] |
| Depression | 6.3 | 5.9 |  | 4.6 | 5.3 |  | 0.975 | 0.329 | 0.02 |  | 0.30 [-0.31, 0.91] |
| Anxiety | 6.2 | 5.1 |  | 6.2 | 5.7 |  | 0.002 | 0.964 | 0.00 |  | 0.00 [-0.61, 0.61] |
| **TICS Screening Scale** | 23.0 | 9.9 |  | 20.7 | 10.8 |  | 0.501 | 0.483 | 0.01 |  | 0.22 [-0.39, 0.82] |
| **TICS Subscales** |  |  |  |  |  |  | **1.238** | **.308** | **0.26** |  |  |
| Work overload | 14.0 | 7.2 |  | 15.1 | 7.5 |  | 0.274 | 0.604 | 0.01 |  | -0.15 [-0.75, 0.46] |
| Social overload | 11.6 | 5.3 |  | 10.3 | 6.0 |  | 0.533 | 0.470 | 0.01 |  | 0.22 [-0.38, 0.83] |
| Pressure to succeed | 17.7 | 6.1 |  | 14.0 | 7.4 |  | 3.148 | 0.084 | 0.07 |  | 0.53 [-0.08, 1.15] |
| Work discontent | 13.6 | 7.4 |  | 13.2 | 7.0 |  | 0.028 | 0.868 | 0.00 |  | 0.05 [-0.55, 0.66] |
| Excessive demands at work | 9.5 | 6.3 |  | 8.9 | 5.1 |  | .094 | 0.761 | 0.00 |  | 0.10 [-0.50, 0.71] |
| Lack of social recognition | 6.3 | 3.4 |  | 6.6 | 4.0 |  | .045 | 0.833 | 0.00 |  | -0.08 [-0.68, 0.53] |
| Social tensions | 12.2 | 6.7 |  | 9.5 | 6.2 |  | 1.756 | 0.193 | 0.04 |  | 0.41 [-0.20, 1.02] |
| Social isolation | 12.2 | 6.5 |  | 9.6 | 4.4 |  | 2.341 | 0.134 | 0.06 |  | 0.46 [-0.15, 1.08] |
| Chronic worrying | 9.5 | 3.7 |  | 8.0 | 4.6 |  | 1.243 | 0.272 | 0.03 |  | 0.35 [-0.26, 0.96] |
| **SCI Stress Total Score** | 52.2 | 24.5 |  | 52.6 | 23.1 |  | 0.004 | 0.948 | 0.00 |  | -0.02 [-0.62, 0.59] |
| **SCI Stress Subscales** |  |  |  |  |  |  | **0.894** | **0.477** | **0.09** |  |  |
| Caused by uncertainty | 18.9 | 9.9 |  | 20.2 | 10.1 |  | 0.183 | 0.671 | 0.01 |  | -0.13 [-0.73, 0.48] |
| Due to excessive demands | 19.7 | 7.9 |  | 18.7 | 7.4 |  | 0.186 | 0.668 | 0.01 |  | 0.13 [-0.48, 0.73] |
| Due to loss | 13.6 | 8.6 |  | 13.7 | 7.1 |  | 0.005 | 0.942 | 0.00 |  | -0.01 [-0.62, 0.59] |
| Stress symptoms | 26.2 | 8.1 |  | 25.1 | 8.2 |  | 0.162 | 0.689 | 0.00 |  | 0.13 [-0.47, 0.74] |
| **SCI Coping Scales** |  |  |  |  |  |  | **0.075** | **0.996** | **0.01** |  |  |
| Positive thinking | 9.8 | 2.9 |  | 9.9 | 3.3 |  | 0.014 | 0.906 | 0.00 |  | -0.03 [-0.64, 0.57] |
| Active stress coping | 10.2 | 3.1 |  | 10.5 | 2.6 |  | 0.082 | 0.777 | 0.00 |  | -0.10 [-0.71, 0.50] |
| Social support | 12.6 | 3.2 |  | 12.9 | 2.8 |  | 0.113 | 0.739 | 0.00 |  | -0.10 [-0.70, 0.51] |
| Support in faith | 7.0 | 2.8 |  | 7.2 | 2.9 |  | 0.066 | 0.799 | 0.00 |  | -0.07 [-0.67, 0.54] |
| Alc. and cig. consumption | 6.0 | 2.9 |  | 5.6 | 1.8 |  | 0.248 | 0.621 | 0.01 |  | 0.16 [-0.44, 0.77] |
| **ERQ Scales** |  |  |  |  |  |  | **1.828** | **0.174** | **0.09** |  |  |
| Reappraisal | 3.6 | 1.8 |  | 4.5 | 1.1 |  | 3.633 | 0.064 | 0.08 |  | -0.60 [-1.22, 0.02] |
| Suppression | 3.3 | 1.6 |  | 3.2 | 1.7 |  | 0.081 | 0.778 | 0.00 |  | 0.06 [-0.55, 0.67] |
| **FNE-K Total Score** | 27.9 | 5.1 |  | 26.7 | 6.2 |  | 0.446 | 0.508 | 0.01 |  | 0.21 [-0.40, 0.81] |
| **LSAS Total Score** | 50.7 | 29.2 |  | 44.5 | 23.3 |  | 0.575 | 0.453 | 0.01 |  | 0.23 [-0.38, 0.84] |
| **LSAS Subscales** |  |  |  |  |  |  | **1.709** | **0.169** | **0.16** |  |  |
| Fear of social interaction | 12.2 | 8.5 |  | 11.1 | 7.9 |  | 0.190 | 0.665 | 0.01 |  | 0.13 [-0.47, 0.74] |
| Fear of performance | 13.1 | 8.3 |  | 12.1 | 7.0 |  | 0.168 | 0.684 | 0.00 |  | 0.13 [-0.48, 0.73] |
| Avoidance of soc. int. | 12.0 | 7.0 |  | 10.9 | 7.0 |  | 0.254 | 0.617 | 0.01 |  | 0.15 [-0.45, 0.76] |
| Avoidance of performance | 13.4 | 7.9 |  | 10.4 | 5.9 |  | 1.955 | 0.170 | 0.05 |  | 0.43 [-0.19, 1.04] |
| **GARS Total Score** | 29.3 | 21.9 |  | 24.3 | 15.0 |  | 0.737 | 0.396 | 0.02 |  | 0.26 [-0.34, 0.87] |
| **GARS Subscales** |  |  |  |  |  |  | **1.616** | **0.191** | **0.15** |  |  |
| Fear in e.s. | 2.1 | 2.5 |  | 1.8 | 2.0 |  | 0.227 | 0.636 | 0.01 |  | 0.13 [-0.48, 0.74] |
| Fear h.l.s.t.s. | 10.2 | 6.7 |  | 8.1 | 4.9 |  | 1.318 | 0.258 | 0.03 |  | 0.35 [-0.26, 0.96] |
| Avoidance in e.s. | 2.9 | 3.4 |  | 2.0 | 2.4 |  | 1.023 | 0.318 | 0.03 |  | 0.30 [-0.31, 0.91] |
| Avoidance h.l.s.t.s. | 9.0 | 6.2 |  | 8.4 | 5.0 |  | 0.117 | 0.735 | 0.00 |  | 0.11 [-0.50, 0.71] |
| **EQ Total Score** | 39.5 | 14.3 |  | 39.1 | 14.7 |  | 0.007 | 0.936 | 0.00 |  | 0.03 [-0.58, 0.63] |
| **EQ Subscales** |  |  |  |  |  |  | **2.085** | **0.118** | **0.14** |  |  |
| Cognitive empathy | 10.4 | 5.9 |  | 12.1 | 6.8 |  | 0.773 | 0.385 | 0.02 |  | -0.26 [-0.87, 0.35] |
| Emotional reactivity | 12.9 | 4.9 |  | 10.8 | 4.2 |  | 2.100 | 0.155 | 0.05 |  | 0.45 [-0.16, 1.07] |
| Social skills | 5.0 | 2.7 |  | 5.5 | 3.0 |  | 0.398 | 0.532 | 0.01 |  | -0.17 [-0.78, 0.44] |
| **IRI Personal distress** | 12.7 | 3.4 |  | 11.6 | 5.5 |  | 0.543 | 0.466 | 0.01 |  | 0.23 [-0.37, 0.84] |
| **IRI Empathy Score^1^** | 55.6 | 14.5 |  | 54.4 | 9.2 |  | 0.111 | 0.741 | 0.00 |  | 0.10 [-0.51, 0.70] |
| **IRI Subscales** |  |  |  |  |  |  | **0.233** | **0.873** | **0.02** |  |  |
| Fantasy | 19.0 | 5.2 |  | 19.1 | 4.5 |  | 0.001 | 0.976 | 0.00 |  | -0.02 [-0.63, 0.59] |
| Perspective taking | 15.4 | 7.2 |  | 15.1 | 5.5 |  | 0.024 | 0.878 | 0.00 |  | 0.05 [-0.56, 0.65] |
| Emphatic concern | 21.3 | 4.6 |  | 20.3 | 4.0 |  | 0.536 | 0.468 | 0.01 |  | 0.23 [-0.38, 0.84] |
| **AQ-k Total Score** | 13.1 | 6.1 |  | 12.9 | 6.0 |  | 0.010 | 0.921 | 0.00 |  | 0.03 [-0.57, 0.64] |
| **AQ-k Subscales** |  |  |  |  |  |  | **3.249** | **0.098** | **0.15** |  |  |
| Soc. int. and spontaneity | 2.8 | 2.6 |  | 4.1 | 2.6 |  | 2.442 | 0.126 | 0.06 |  | -0.49 [-1.11, 0.12] |
| Fantasy and imagination | 4.9 | 2.3 |  | 3.7 | 2.5 |  | 2.288 | 0.138 | 0.05 |  | 0.49 [-0.13, 1.10] |
| Comm. and reciprocity | 5.4 | 2.6 |  | 5.1 | 3.0 |  | 0.126 | 0.725 | 0.00 |  | 0.10 [-0.50, 0.71] |
| **NEO-FFI Scales** |  |  |  |  |  |  | **0.552** | **0.736** | **0.07** |  |  |
| Neuroticism | 2.2 | 0.8 |  | 1.9 | 1.1 |  | 0.652 | 0.424 | 0.02 |  | 0.30 [-0.31, 0.91] |
| Extraversion | 2.0 | 0.7 |  | 2.1 | 0.6 |  | 0.040 | 0.843 | 0.00 |  | -0.15 [-0.76, 0.46] |
| Openness | 2.3 | 0.8 |  | 2.4 | 0.6 |  | 0.010 | 0.919 | 0.00 |  | -0.14 [-0.75, 0.47] |
| Agreeableness | 2.7 | 0.7 |  | 2.5 | 0.9 |  | 0.621 | 0.435 | 0.02 |  | 0.24 [-0.37, 0.85] |
| Conscientiousness | 2.8 | 0.7 |  | 2.6 | 0.6 |  | 0.447 | 0.507 | 0.01 |  | 0.30 [-0.31, 0.91] |

Notes: *BDI-II* Beck Depression Inventory II; *STAI-T* Trait scale of the State-Trait Anxiety Inventory; *Mini-SCL* Mini Symptom Checklist; *GSI* Global Severity Index; *TICS* Trier Inventory for Chronic Stress; *SCI* Stress and Coping Inventory; *ERQ* Emotion Regulation Questionnaire; *Alc.* Alcohol; *cig.* cigarettes; *FNE-K* Fear of Negative Evaluation Scale–short version; *LSAS* Liebowitz Social Anxiety Scale; *soc. int.* social interaction; *GARS* Gaze Anxiety Rating Scale; *e.s.* everyday situations; *h.l.s.t.s.* high level social threat situations; *EQ* Empathy Quotient; *IRI* Interpersonal Reactivity Index; *AQ-k* Autism Spectrum Quotient short version; *comm.* communication; *NEO-FFI* NEO-Five-Factor Inventory

^1^ Sum of the IRI subscales except personal distress subscale

**Table S3.** Group differences in psychometric and psychopathological characteristics of women with TXS and controls

|  | TXS (n=44) | | Controls (n=50) | | |  | **MANOVA**/ANOVA | | |  |  |
| --- | --- | --- | --- | --- | --- | --- | --- | --- | --- | --- | --- |
|  | ***M*** | ***SD*** |  | ***M*** | ***SD*** |  | ***F*** | ***p*** | ***η^2^p*** |  | ***d* [95 %-CI]** |
| **BDI-II Total Score** | 14.9 | 11.5 |  | 12.6 | 9.3 |  | 1.216 | 0.273 | 0.01 |  | 0.22 [−0.19, 0.63] |
| **STAI-T Total Score** | 45.7 | 11.7 |  | 45.3 | 10.6 |  | 0.025 | 0.876 | 0.00 |  | 0.04 [−0.37, 0.44] |
| **Mini-SCL GSI** | 18.5 | 13.9 |  | 13.1 | 11.6 |  | 4.169 | 0.044 | 0.04 |  | 0.42 [0.01, 0.83] |
| **Mini-SCL Subscales** |  |  |  |  |  |  | **2.519** | **0.063** | **0.08** |  |  |
| Somatization | 6.4 | 5.3 |  | 3.8 | 3.9 |  | 7.407 | 0.008 | 0.08 |  | 0.56 [0.15, 0.97] |
| Depression | 5.7 | 5.8 |  | 4.7 | 4.9 |  | 2.888 | 0.093 | 0.03 |  | 0.19 [−0.22, 0.59] |
| Anxiety | 6.4 | 5.5 |  | 4.6 | 5.1 |  | 0.851 | 0.359 | 0.01 |  | 0.34 [−0.07, 0.75] |
| **TICS Screening Scale** | 21.9 | 10.2 |  | 22.7 | 10.0 |  | 0.122 | 0.727 | 0.00 |  | -0.08 [-0.48, 0.33] |
| **TICS Subscales** |  |  |  |  |  |  | **2.175** | **0.032** | **0.19** |  |  |
| Work overload | 14.4 | 7.2 |  | 15.9 | 7.1 |  | 1.085 | 0.300 | 0.01 |  | -0.21 [-0.61, 0.20] |
| Social overload | 10.9 | 5.6 |  | 12.1 | 5.5 |  | 1.189 | 0.278 | 0.01 |  | -0.21 [-0.62, 0.19] |
| Pressure to succeed | 15.8 | 6.9 |  | 16.3 | 6.8 |  | 0.098 | 0.755 | 0.00 |  | -0.07 [-0.48, 0.33] |
| Work discontent | 13.6 | 7.2 |  | 12.1 | 5.7 |  | 1.262 | 0.264 | 0.01 |  | 0.23 [-0.18, 0.64] |
| Excessive demands at work | 9.2 | 5.5 |  | 7.9 | 4.7 |  | 1.622 | 0.206 | 0.02 |  | 0.25 [-0.15, 0.66] |
| Lack of social recognition | 6.6 | 3.7 |  | 7.4 | 3.8 |  | 1.218 | 0.273 | 0.01 |  | -0.21 [-0.62, 0.19] |
| Social tensions | 10.8 | 6.4 |  | 7.6 | 5.2 |  | 6.863 | 0.010 | 0.07 |  | 0.55 [ 0.14, 0.96] |
| Social isolation | 11.0 | 5.9 |  | 8.9 | 5.5 |  | 3.067 | 0.083 | 0.03 |  | 0.37 [-0.04, 0.77] |
| Chronic worrying | 8.8 | 4.3 |  | 8.7 | 3.9 |  | 0.020 | 0.887 | 0.00 |  | 0.02 [-0.38, 0.43] |
| **SCI Stress Total Score** | 52.8 | 23.0 |  | 56.0 | 20.0 |  | 0.503 | 0.480 | 0.01 |  | -0.15 [-0.55, 0.26] |
| **SCI Stress Subscales** |  |  |  |  |  |  | **4.155** | **0.004** | **0.16** |  |  |
| Caused by uncertainty | 19.7 | 9.7 |  | 22.4 | 8.2 |  | 2.016 | 0.159 | 0.02 |  | -0.30 [-0.71, 0.11] |
| Due to excessive demands | 19.2 | 7.5 |  | 21.5 | 7.5 |  | 2.182 | 0.143 | 0.02 |  | -0.30 [-0.71, 0.10] |
| Due to loss | 13.9 | 7.7 |  | 12.1 | 5.9 |  | 1.675 | 0.199 | 0.02 |  | 0.26 [-0.14, 0.67] |
| Stress symptoms | 25.9 | 8.0 |  | 25.6 | 7.0 |  | 0.030 | 0.862 | 0.00 |  | 0.04 [-0.37, 0.44] |
| **SCI Coping Scales** |  |  |  |  |  |  | **2.518** | **0.035** | **0.13** |  |  |
| Positive thinking | 9.8 | 3.1 |  | 9.4 | 2.2 |  | 0.713 | 0.401 | 0.01 |  | 0.15 [-0.26, 0.55] |
| Active stress coping | 10.4 | 2.8 |  | 10.4 | 2.7 |  | 0.003 | 0.959 | 0.00 |  | 0.00 [-0.41, 0.41] |
| Social support | 12.6 | 3.2 |  | 12.4 | 3.2 |  | 0.103 | 0.749 | 0.00 |  | 0.06 [-0.34, 0.47] |
| Support in faith | 7.3 | 3.0 |  | 7.1 | 2.4 |  | 0.078 | 0.781 | 0.00 |  | 0.07 [-0.33, 0.48] |
| Alc. and cig. consumption | 5.8 | 2.3 |  | 8.0 | 3.4 |  | 12.895 | .0005 | 0.12 |  | -0.74 [-1.16, -0.32] |
| **ERQ Scales** |  |  |  |  |  |  | **1.173** | **0.314** | **0.03** |  |  |
| Reappraisal | 4.0 | 1.5 |  | 4.4 | 1.0 |  | 2.156 | 0.145 | 0.02 |  | -0.32 [-0.72, 0.09] |
| Suppression | 3.3 | 1.6 |  | 3.5 | 1.1 |  | 0.195 | 0.660 | 0.00 |  | -0.15 [-0.55, 0.26] |
| **FNE-K Total Score** | 27.5 | 5.6 |  | 27.1 | 5.5 |  | 0.146 | 0.703 | 0.00 |  | 0.03 [-0.37, 0.44] |
| **LSAS Total Score** | 46.9 | 25.7 |  | 36.7 | 25.2 |  | 3.720 | 0.057 | 0.04 |  | 0.40 [-0.01, 0.81] |
| **LSAS Subscales** |  |  |  |  |  |  | **1.096** | **0.363** | **0.05** |  |  |
| Fear of social interaction | 11.4 | 8.0 |  | 8.2 | 6.9 |  | 2.779 | 0.099 | 0.03 |  | 0.43 [ 0.02, 0.84] |
| Fear of performance | 12.5 | 7.4 |  | 10.0 | 6.9 |  | 1.931 | 0.168 | 0.02 |  | 0.35 [-0.06, 0.76] |
| Avoidance of soc. int. | 11.2 | 6.9 |  | 8.8 | 6.4 |  | 4.425 | 0.038 | 0.05 |  | 0.36 [-0.05, 0.77] |
| Avoidance of performance | 11.6 | 6.9 |  | 9.7 | 7.2 |  | 3.139 | 0.080 | 0.03 |  | 0.27 [-0.14, 0.67] |
| **GARS Total Score** | 26.7 | 18.3 |  | 22.1 | 21.1 |  | 1.239 | 0.269 | 0.01 |  | 0.23 [-0.18, 0.64] |
| **GARS Subscales** |  |  |  |  |  |  | **0.706** | **0.590** | **0.03** |  |  |
| Fear in e.s. | 1.9 | 2.2 |  | 1.8 | 2.5 |  | 0.035 | 0.852 | 0.00 |  | 0.04 [-0.36, 0.45] |
| Fear h.l.s.t.s. | 9.1 | 5.7 |  | 7.4 | 6.3 |  | 1.824 | 0.180 | 0.02 |  | 0.07 [-0.34, 0.47] |
| Avoidance in e.s. | 2.4 | 2.8 |  | 2.2 | 3.2 |  | 0.067 | 0.796 | 0.00 |  | 0.28 [-0.13, 0.69] |
| Avoidance h.l.s.t.s. | 8.7 | 5.5 |  | 7.2 | 6.3 |  | 1.489 | 0.225 | 0.02 |  | 0.25 [-0.16, 0.66] |
| **EQ Total Score** | 39.4 | 14.1 |  | 40.3 | 12.0 |  | 0.113 | 0.737 | 0.00 |  | -0.07 [-0.47, 0.34] |
| **EQ Subscales** |  |  |  |  |  |  | **0.154** | **0.927** | **0.01** |  |  |
| Cognitive empathy | 11.2 | 6.4 |  | 11.1 | 5.4 |  | 0.015 | 0.902 | 0.00 |  | 0.02 [-0.39, 0.42] |
| Emotional reactivity | 11.9 | 4.6 |  | 12.2 | 4.2 |  | 0.105 | 0.747 | 0.00 |  | -0.07 [-0.47, 0.34] |
| Social skills | 5.2 | 2.8 |  | 5.4 | 2.4 |  | 0.206 | 0.651 | 0.00 |  | -0.08 [-0.48, 0.33] |
| **IRI Personal distress** | 12.1 | 4.5 |  | 10.2 | 4.4 |  | 4.385 | 0.039 | 0.05 |  | 0.42 [ 0.01, 0.83] |
| **IRI Empathy Score^1^** | 54.7 | 11.7 |  | 55.7 | 12.2 |  | 0.162 | 0.688 | 0.00 |  | -0.08 [-0.49, 0.32] |
| **IRI Subscales** |  |  |  |  |  |  | **1.459** | **0.231** | **0.05** |  |  |
| Fantasy | 18.8 | 4.8 |  | 18.1 | 5.4 |  | 0.485 | 0.488 | 0.01 |  | 0.14 [-0.27, 0.54] |
| Perspective taking | 15.1 | 6.2 |  | 17.0 | 5.8 |  | 2.402 | 0.125 | 0.03 |  | -0.31 [-0.72, 0.09] |
| Emphatic concern | 20.8 | 4.2 |  | 20.6 | 4.3 |  | 0.039 | 0.844 | 0.00 |  | 0.05 [-0.36, 0.45] |
| **AQ-k Total Score** | 12.9 | 5.9 |  | 9.7 | 5.7 |  | 7.111 | 0.009 | 0.07 |  | 0.55 [ 0.13, 0.96] |
| **AQ-k Subscales** |  |  |  |  |  |  | **3.007** | **0.034** | **0.09** |  |  |
| Soc. int. and spontaneity | 3.5 | 2.6 |  | 2.8 | 2.4 |  | 1.592 | 0.210 | 0.02 |  | 0.28 [-0.13, 0.69] |
| Fantasy and imagination | 4.2 | 2.4 |  | 3.2 | 2.6 |  | 3.676 | 0.058 | 0.04 |  | 0.40 [-0.01, 0.80] |
| Comm. and reciprocity | 5.2 | 2.7 |  | 3.7 | 2.3 |  | 9.034 | 0.003 | 0.09 |  | 0.60 [ 0.18, 1.01] |
| **NEO-FFI Scales** |  |  |  |  |  |  | **0.873** | **0.503** | **0.05** |  |  |
| Neuroticism | 2.1 | 1.0 |  | 1.9 | 0.9 |  | 1.505 | 0.223 | 0.02 |  | 0.25 [-0.15, 0.66] |
| Extraversion | 2.0 | 0.7 |  | 2.2 | 0.6 |  | 2.344 | 0.129 | 0.03 |  | -0.31 [-0.71, 0.10] |
| Openness | 2.4 | 0.7 |  | 2.3 | 0.9 |  | 0.411 | 0.523 | 0.00 |  | 0.12 [-0.28, 0.53] |
| Agreeableness | 2.6 | 0.8 |  | 2.7 | 0.9 |  | 0.291 | 0.591 | 0.00 |  | -0.12 [-0.53, 0.29] |
| Conscientiousness | 2.7 | 0.6 |  | 2.9 | 0.7 |  | 1.479 | 0.227 | 0.02 |  | -0.25 [-0.66, 0.16] |

Notes: *BDI-II* Beck Depression Inventory II; *STAI-T* Trait scale of the State-Trait Anxiety Inventory; *Mini-SCL* Mini Symptom Checklist; *GSI* Global Severity Index; *TICS* Trier Inventory for Chronic Stress; *SCI* Stress and Coping Inventory; *ERQ* Emotion Regulation Questionnaire; *Alc.* Alcohol; *cig.* cigarettes; *FNE-K* Fear of Negative Evaluation Scale–short version; *LSAS* Liebowitz Social Anxiety Scale; *soc. int.* social interaction; *GARS* Gaze Anxiety Rating Scale; *e.s.* everyday situations; *h.l.s.t.s.* high level social threat situations; *EQ* Empathy Quotient; *IRI* Interpersonal Reactivity Index; *AQ-k* Autism Spectrum Quotient short version; *comm.* communication; *NEO-FFI* NEO-Five-Factor Inventory

^1^ Sum of the IRI subscales except personal distress subscale
